# Vitamin D status, vitamin D receptor, CYP2R1, and CYP24A1 profiles in children

**DOI:** 10.3389/fnut.2024.1394367

**Published:** 2024-06-07

**Authors:** Anggraini Iriani, Andhika Rachman, Marsya Kaila Fatina, Rizka Kurnia Gemilang, Andi Trisnandi, Fiona Valerie Muskananfola, Media Fitri Isma Nugraha

**Affiliations:** ^1^Department of Clinical Pathology, Faculty of Medicine Yarsi University-Yarsi Hospital, Jakarta, Indonesia; ^2^Department of Hematology Oncology, Cipto Manguskusumo Hospital, Jakarta, Indonesia; ^3^Faculty of Medicine, Andalas University, Padang, Indonesia; ^4^Faculty of Medicine, Yarsi University, Jakarta, Indonesia; ^5^Genomics Laboratory, Bunda Hospital, Jakarta, Indonesia; ^6^Faculty of Medicine, University of Indonesia, Jakarta, Indonesia; ^7^Research Center for Pharmaceutical Ingredients and Traditional Medicine – National Research and Innovation Agency, Cibinong, Indonesia

**Keywords:** vitamin D, vitamin D receptor, CYP2R1, CYP24A1, pediatric, adolescence

## Abstract

**Introduction:**

Vitamin D plays a major role in the musculoskeletal and immune system. Understanding the comprehensive mechanism of vitamin D receptors and the enzyme of vitamin D induction (CYP2R1) and inhibition (CYP24A1) in its metabolism is interesting. This study aims to understand vitamin D metabolism in Indonesian pediatrics, specifically in Jakarta, which has abundant sun exposure.

**Methodology:**

A cross-sectional study with comparative, correlative, and multivariate analysis on vitamin D, vitamin D receptor, CYP2R1, and CYP24A1 levels was conducted on 46 children with no known morbidity.

**Result:**

Subjects were mostly male (52.2%), age group of 2–6 years (34.8%), and had sufficient vitamin D status (43.5%, median 27.55 ng/mL). Age was found to have a negative correlation with vitamin D levels (*p* < 0.001; *r* = −0.625) and CYP2R1 (*p* = 0.035; *r* = −0.311). Significant positive associations were found between CYP24A1 and CYP2R1 (*p* = 0.046; *r* = 0.296). Participants aged 0–2 are more likely to have a higher level of vitamin D status compared to those aged >2 years (OR 42.092, 95% CI [4.532–390.914], *p* = 0.001). VDR levels were significantly lower in insufficient vitamin D levels than in the sufficient group (*p* = 0.018). VDR and vitamin D status had a positive relation (OR 7.023, 95% CI [1.864–26.453], *p* = 0.004).

**Conclusion:**

Vitamin D levels decrease with the increase in age. Vitamin D receptor level has an inline-level progression with vitamin D level. CYP2R1 and CYP24A1 suggest a directly proportional relationship. Vitamin D screening and supplementation in children older than 2 years old are suggested.

## Introduction

Vitamin D is categorized into vitamin D deficiency (<20 ng/mL), insufficiency (21–30 ng/mL), and sufficiency (>30 ng/mL). The prevalence of vitamin D has been widely studied among continents (1, 2). Vitamin D deficiency is not limited to non-tropical, but also in tropical countries, which have abundant sunlight (3, 4). Vitamin D is a principal factor in bone and neuron health, immunity, cancer, and cardiovascular disease (5). Vitamin D deficiency in children increases the risk of rachitis, seizure, hypocalcemia, and delayed tooth growth. In adolescence, vitamin D deficiency is associated with musculoskeletal pain, weak muscle tone, lower extremity deformity, and increased risk of acute or chronic diseases (6). Furthermore, vitamin D deficiency is related to obesity, cardiovascular risk, insulin resistance, beta cell dysfunction, autoimmune diseases, and cancer. Factors contributing to vitamin D levels include sunlight exposure, skin pigmentation, diet, and vitamin D supplementation (7, 8).

In Southeast Asia, 1 in 2 neonates and more than 60% of adolescents have vitamin D deficiency. In Indonesia and Thailand, 9 of 10 neonates have vitamin D deficiency (6). The percentage of Indonesian children with insufficient and inadequate vitamin D is relatively higher compared to Malaysia, Thailand, and Vietnam. Research on vitamin D deficiency in Indonesia has not been done widely. However, research in 2015 of children aged 2–12 years old in 47 Indonesian districts showed that 44% was insufficient, 50.3% was inadequate, and only 5.6% was sufficient in vitamin D (9). Abboud et al. stated that factors affecting vitamin D 25(OH)D level in circulation, such as cholesterol synthesis, hydroxylation, and vitamin D transport, is mediated by cytochrome P450 or CYP450, including CYP2R1 and CYP24A1 (10). The metabolism of vitamin D seems to be affected by race or ethnicity. Prior studies indicated that racial and ethnic differences are evident in the markers of vitamin D metabolism, which can be attributed, at least in part, to genetic heritage (11). In addition, other factors including gene polymorphism and locus variation, vitamin D hydroxylation epigenetic regulation (Deoxyribonucleic acid or DNA methylation), calcium intake, and the density of fat and muscle tissue also contribute to vitamin D levels (12, 13).

Provitamin D3 in the skin (7-dehydrocholesterol) and D2 (ergosterol) from the diet are the main sources of vitamin D. Ultraviolet B (UVB) from sunlight is required to convert 7-dehydrocholesterol into cholecalciferol (vitamin D3). Ergosterol will be converted into ergocalciferol (vitamin D2) (14). The enzymatic conversion in the liver is mediated by CYP2R1, which helps the formation of calcidiol (25(OH)D) (15). Following interaction with 1α hydroxylase in the renal, calcidiol becomes its active form, 1,25(OH)2D3 (calcitriol). Calcitriol binds to vitamin D receptors (VDR) to function in each organ (2). Once vitamin D concentration is high, the synthesis is reduced by inducing the CYP24A1 activity which inactivates and accelerates the catabolism of calcitriol (16).

Although the importance of vitamin D in children and the influence of demographic background on vitamin D levels has been widely acknowledged, there has been no study regarding vitamin D, VDR, CYP2R1, and CYP24A1 in Indonesian pediatrics. This study aims to analyze the relation of vitamin D levels with VDR and CYP450 (CYP2R1 and CYP24A1) levels in Indonesian children.

## Materials and methods

### Study design

This research is a cross-sectional study conducted in Bunda Woman and Children Hospital, Jakarta, Indonesia. The subjects included in this study were 46 subjects. Inclusion criteria were < 18 years old; without comorbidities and chronic disease, which was determined based on physical examination and complete blood test. Those who declined research participation were excluded. Obtained data included gender, age, vitamin D, VDR, CYP2R1, and CYP24A1. The measured variables, vitamin D, VDR, CYP2R1, and CYP24A1, were analyzed based on age group: 0–2 years old, 2–6 years old, and 6–18 years old.

The sample size was calculated using the formula of correlative analytic research and MedCalc application (17). This research used a 95% confidence interval (CI), β of 10%, and a correlation coefficient of 0.5. Therefore, the applied Zα and Zβ were 1.96 and 1.282, respectively (18).


$$n={\left[\frac{\left(Z\alpha +Z\beta \right)}{0.5\;\ln\left(\frac{1+r}{1-r}\right)}\right]}^{2}+3={\left[\frac{\left(1.96+1.282\right)}{0.5\;\ln\left(\frac{1+0.5}{1-0.5}\right)}\right]}^{2}+3=37.84\approx 38$$

### Measurement

Vitamin D level measurement was completed by examining 25 (OH) D serum levels. A total of 3–5 mL of the sample’s blood was placed in a serum tube from a cuffed venous sample. The tool used for this examination was Roche Diagnostics’ Cobas e411 which was a combination of competitive immunoassay and enzyme-catalyzed chemiluminescence detection (CLIA test). Examination of VDR and CYP24A1 serum levels was carried out using the ELISA method and reagen from Elabscience, USA (Catalog No: E-EL-H2043 and E-EL-H0377). ELISA method was also used for CYP2R1 serum levels examination reagen from Abebio, China (Catalog No: AE48038HU). All procedures were carried out based on the manufacturer’s recommendation.

### Statistical analysis

Data were analyzed using Statistical Product and Service Solutions (SPSS) application system version 26.0. The data normality test was carried out on all subjects using Shapiro–Wilk (n < 50). Data were normally distributed if the *p*-value >0.05. Mean and standard deviation were used in normally distributed data; otherwise, median and interquartile range (IQR) were applied. Bivariate comparative analysis was carried out using an independent T-test (≤2 groups) or One Way Anova (>2 groups) in normally distributed data. Meanwhile, the Mann–Whitney test (≤2 groups) or the Kruskal-Wallis test (>2 groups) was used in abnormal data distribution. The correlation analysis between subject characteristics (gender, age, and vitamin D status), vitamin D levels, VDR, CYP2R1, and CYP24A1 levels was carried out. Correlation tests were completed using Pearson, Spearman, or Eta correlation, depending on the variable type. *p*-values <0.05 were considered statistically significant. Significant findings on correlation tests were further analyzed using *post hoc* analysis. Multivariate analysis of vitamin D status was conducted using ordinal regression analysis and generalized linear model. The factors included were sex and age group. The covariates included were the levels of VDR, CYP2R1, and CYP24A1.

### Ethical approval

The aim of the study had been explained and assent consent had been obtained from all subjects. This research was approved based on the Helsinki Declaration by the Ethics Commission of the Faculty of Medicine, YARSI University (No: 030/KEP-UY/EA.20/II/2023).

## Results

A total of 46 subjects(<18 years old) data, collected from Bunda Woman and Children Hospital, Jakarta, Indonesia, were included in this study. Subjects were mostly male (52.2%), age group of 2–6 (34.8%), and had sufficient vitamin D status (43.5%). Subject demographic based on vitamin D status (Table 1), showed that 50% of males and females had sufficient and insufficient vitamin D status, respectively. Based on age, the age group of 0–2 years mostly had sufficient vitamin D status (86.7%), meanwhile, the 2–18 years had insufficient vitamin D status.

**Table 1:** Subject demographic based on vitamin D status.

	*n* (%)	Vitamin D status		
		Deficient (%)	Insufficient (%)	Sufficient (%)
Sex				
Male	24 (52.9)	5 (20.8)	7 (29.2)	12 (50.0)
Female	22 (47.8)	3 (13.6)	11 (50.0)	8 (36.4)
Age (years)				
0 to <2	15 (32.6)	0 (0.0)	2 (13.3)	13 (86.7)
2 to <6	16 (34.8)	3 (18.8)	7 (43.7)	6 (37.5)
6–18	15 (32.6)	5 (33.3)	9 (60.0)	1 (6.7)

**Figure 1:**
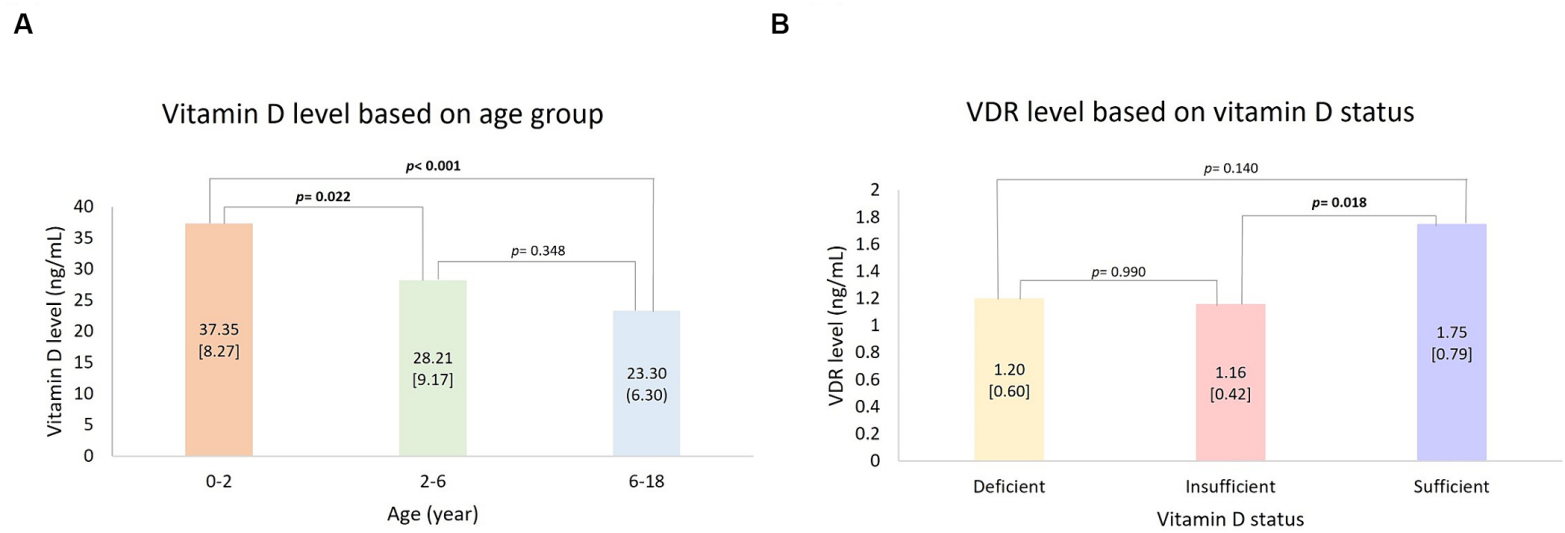
*Post hoc* analysis: **(A)** Vitamin D level based on age group and **(B)** VDR level based on vitamin D status. Bold value indicates a statistically significant comparison.

The vitamin D level median among the subjects was 27.55 ng/mL. Significant differences were found in vitamin D levels based on age (*p* < 0.001). The highest vitamin D was found in 0–2 years old, 37.35 [8.27] ng/mL, and the lowest in 6–18 years old, 23.30 (6.30) ng/mL. Mann Whitney *post hoc* test was conducted and resulted in significantly higher vitamin D levels in age group 0–2 years in comparison to 2–6 years (*p* = 0.022) and 6–17 years (*p* < 0.001) (Figure 1A). VDR level was significantly different based on vitamin D status (*p* = 0.031) with the highest mean found in the sufficient group (1.75 [0.787] ng/mL). The analysis was followed with the Games-Howell *post hoc* test and resulted in VDR levels in sufficient vitamin D status being higher in comparison to those in insufficient group (Figure 1B; *p* = 0.018). There was no significant value comparison of CYP2R1 and CYP24A1 based on gender, age, and vitamin D status. Subject characteristics and the comparative test results are presented in Table 2.

**Table 2:** Subject characteristics with vitamin D, VDR, CYP2R1, and CYP24A1 levels.

Variables	*n* = 46 (%)	Vitamin D (ng/mL)	*p*-value	VDR (ng/mL)	*p*-value	CYP2R1 (ng/mL)	*p*-value	CYP24A1 (ng/mL)	*p*-value
Sex									
Male	24 (52.2)	30.26 [8.97]	0.645	1.50 [0.67]	0.441	20.95 (28.91)	0.947	0.48 [0.25]	0.787
Female	22 (47.8)	28.93 [10.45]		1.13 (0.55)		19.89 (27.01)		0.50 [0.21]	
Age (years)									
0 to <2	15 (32.6)	37.35 [8.27]	<0.001*	1.46 [0.69]	0.286	22.86 (78.02)	0.110	0.40 (0.24)	0.207
2 to <6	16 (34.8)	28.21 [9.17]		1.60 [0.76]		24.20 [14.19]		0.56 [0.17]	
6–18	15 (32.6)	23.30 (6.30)		1.21 [0.57]		15.91 (20.15)		0.45 [0.29]	
Vitamin D status									
Deficient	8 (17.4)	18.25 [1.88]	<0.001*	1.20 [0.60]	0.031*	20.37 [8.19]	0.122	0.54 [0.25]	0.710
Insufficient	18 (39.1)	24.67 [2.75]		1.16 [0.42]		15.66 (28.73)		0.51 [0.23]	
Sufficient	20 (43.5)	37.70 (8.88)		1.75 [0.79]		22.27 (34.63)		0.46 [0.24]	

Mean [SD] or Median (IQR); IQR, interquartile range; SD, standard deviation; VDR, vitamin D receptor; CYP, cytochrome.

*p*-value denotes the comparison analysis between variables (sex, age, and vitamin D status) and the level of vitamin D, VDR, CYP2R1, and CYP24A1. *p < 0.05 denotes statistical significance.

**Figure 2:**
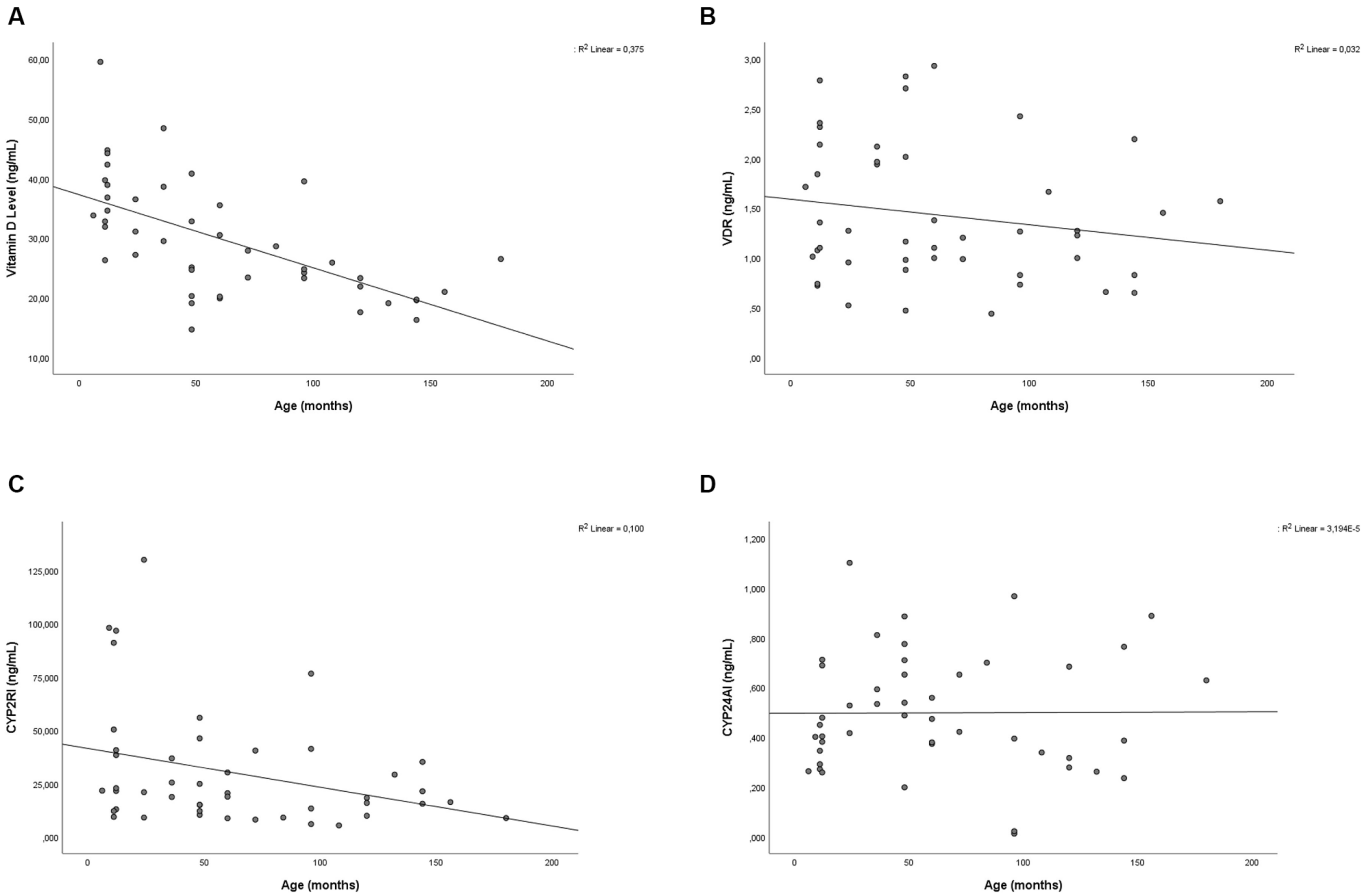
Scatter plot showing the relationship between age with **(A)** vitamin D, **(B)** VDR, **(C)** CYP2R1, and **(D)** CYP24A1 level.

Correlation bivariate analysis was conducted based on subject characteristics. Age was found to have a negative correlation with vitamin D levels (*p* < 0.001; *r* = −0.625) and CYP2R1 (*p* = 0.035; *r* = −0.311). The scatterplot of the correlation between age and vitamin D, VDR, CYP2R1, and CYP24A1 is provided in Figure 2. VDR and vitamin D status had a positive correlation (*p* = 0.013; *r* = 0.363). There was no significant association finding on CYP24A1 based on subject characteristics. Between measured variables, which were vitamin D, VDR, CYP2R1, and CYP24A1, a bivariate correlation test was carried out. Significant positive correlations were found between vitamin D level and VDR level (*p* = 0.006; *r* = 0.399) and in CYP24A1 and CYP2R1 (*p* = 0.046; *r* = 0.296). Complete correlation test results are depicted in Table 3.

**Table 3:** Correlation analysis between vitamin D level, VDR, CYP2R1, CYP24A1, and subject characteristics.

*n* = 46	Vitamin D (ng/mL)		VDR (ng/mL)		CYP2R1 (ng/mL)		CYP24A1 (ng/mL)	
	*r*	*p*	*r*	*p*	*r*	*p*	*r*	*p*
Sex	0.07	0.645	0.108	0.473	0.003	0.985	0.041	0.787
Age	−0.625	<0.001*	−0.14	0.354	−0.311	0.035*	−0.004	0.979
Vitamin D status	0.924	<0.001*	0.363	0.013*	0.259	0.083	−0.110	0.468
VDR	0.399	0.006*						
CYP2R1	0.185	0.219	−0.172	0.252				
CYP24A1	−0.103	0.496	−0.128	0.398	0.296	0.046*		

*p < 0.05 denotes statistical significance.

The ordinal multivariate analysis was conducted to examine the association between vitamin D status, sex, age, and VDR. CYP2R1, and CYP24A1 level. The ordinal regression analysis showed that the included independent variables influence vitamin D status up to 62.0% (pseudo *R*^2^ = 0.620). The generalized linear model suggests that VDR (OR 7.023, 95% CI [1.864–26.453], *p* = 0.004) is positively associated with vitamin D level. The estimate from Table 4 suggests that participants aged 0–2 are more likely to have a higher level of vitamin D status compared to participants aged > 2 years (OR 42.092, 95% CI [4.532–390.914], *p* = 0.001). There were no significant associations between the level of CYP2R1 and CYP24A1 and sex. The multivariate analysis results are described in Table 4.

**Table 4:** Multivariate analysis of vitamin D status factors.

	OR	95% CI		*p*-value	pseudo R^2^
		Lower	Upper		
**Parameter**					0.620
Sex – male	0.549	0.120	2.520	0.441	
Age (years) – 0 to 2 years old	42.092	4.532	390.914	0.001*	
Age (years) – 2 to 6 years old	2.262	0.417	12.274	0.344	
VDR (ng/mL)	7.023	1.864	26.453	0.004*	
CYP2RI (ng/mL)	1.037	0.999	1.077	0.057	
CYP24AI (ng/mL)	0.149	0.006	3.716	0.246	

OR, odds ratio; CI, confidence interval; *p < 0.05 denotes statistical significance.

## Discussion

This study aims to understand the relationship between vitamin D level, VDR, CYP2R1, and CYP24A1. Vitamin D deficiency is common in South East Asia pediatrics (6). Previous research in an Indonesian pediatric sample aged 7–12 years in Jakarta indicated that most children were insufficient in vitamin D (19). Other research showed that in the age group of 2.0–12.9 years vitamin D mean was inadequate (9). However, the current research indicated that most of the participants had sufficient vitamin D status (43.5%). This discrepancy can be explained by different age groups of subjects. Our subjects had a wider age range (<18 years) and were normally distributed between each age range. Younger age is known to have higher vitamin D levels, which might elevate the whole sample’s mean (20). Furthermore, the global consensuses, for example, World Health Organization (WHO), Centers for Disease Control and Prevention (CDC), and Paediatric Association, on vitamin D supplementation as prophylaxis have been applied widely. These guidelines focus on preventing rickets, infections, and other chronic disease (21). The recommended vitamin D supplementation dose in infants is 400 IU. However, vitamin D dose recommendations ideally differ between age groups and predispose factors, ranging from 400–4,000 IU/day or 10–100 μg/day (22).

Vitamin D levels were significantly higher in the age group 0–2 years in comparison to the age group of 2–6 years and 6–18 years. The age group of 0–2 years was found to be one of the vitamin D predictors. Age 0–2 years were found to have the highest vitamin D level. Vitamin D deficiency was prevalent in children aged 6–12 years in Busan with a mean of 14.86 ± 3.20 ng/mL (23). Other studies showed that in comparison to preschoolers, a considerably higher percentage of elementary school students and teenagers fell into the category of vitamin D deficiency and overall low vitamin D (20). In Indonesia, a previous study showed that younger children had better vitamin D mean. Ernawati et al. found that vitamin D in the age group of 2.0–2.9 (54 ± 2.3 nmoL/L) was higher than in the age group of 9.0–12.9 (50.3 ± 1.4 nmoL/L) (9). The age group of 6–18 years was found to have the lowest vitamin D level in this study. It can be partially explained by the increasing vitamin D demand during pubertal maturation as vitamin D is required for rapid linear growth as well as bone accrual (24). As the finding indicated that vitamin D decreased in children >2 years old, it raised the question of whether children’s vitamin D levels should be tested as early as 2 years old. A previous study with similar findings also highlighted the need for vitamin D supplementation in children >2 years old (25).

The high level of vitamin D in children under 2 years old might be related to, although was not explored in this study, dietary and behavioral aspects. The dietary intake in children under 2 years old differ from other age groups as in this age, breast milk is given. The World Health Organization recommended exclusive breastfeeding on the first 6 month of life, and continues up to 2 years old with appropriate complimentary food (26). The vitamin D level of breast milk remains rather steady even after prolonged breastfeeding. The breastmilk content is dependent on the level of vitamin D in the mother and, as a result, rises when breastfeeding women receive pharmaceutical vitamin D supplements (27). However, there has been a study showing that exclusively breastfeeding is a risk factor for vitamin D deficiency in children under 6 months old. The vitamin D deficiency at birth in Indonesian infants by 6 months old, specifically among exclusively breastfed, was improved by the Indonesian culture of infant sunbathing (28). This study showed that with the increase of age, the vitamin D level decreased. It is in line with a previous study in Bahrain which indicated a negative correlation between vitamin D levels and age (20). Biological factors such as body composition shifting occur as children grow. Chen et al. found that in preschool children, the fat mass index decreased with age, as the fat-free mass index, consisting of skeletal muscle or lean mass, increased (29). Vitamin D is important to muscle regeneration, especially after muscle damage (30). In addition to inadequate vitamin D supplementation, the increased demand for muscle mass and fibers lowers the vitamin D level (31). Certain social and behavioral factors have been linked to vitamin D deficiency. In Southeast Asia, fair skin is considered the common beauty standard. These factors lead to sun avoidance behaviors including the usage of covered-up garments and the tendency of being indoors (6). Other behaviors that are related to lower levels of vitamin D due to lesser sun exposure include more indoor activities and increased screen time (9, 32, 33). VDR is a member of the steroid hormone receptor family and is involved in transcription in a variety of cell types. It can be found in the membrane, mitochondria, and nucleus of cells (34). Based on vitamin D status, VDR levels were significantly higher in the sufficient group in comparison to the insufficient group. Those who had sufficient vitamin D status had the highest VDR level. The relation of vitamin D, in particular calcitriol, and VDR has been established (2). The unbound VDR is mostly found in the nucleus and slightly distributed on the membrane (35). The distribution is related to VDR mechanism of work. The genomic effect is through the binding of the high-affinity intracellular VDR and calcitriol which forms a heterodimer with the retinoid X receptor. The target organs’ essential gene expression is regulated as a result of the binding of heterodimer to vitamin D responsive elements (VDRE) in DNA once it reaches the cell nucleus (5). As VDR is essential in initiating vitamin D function, and vice versa, vitamin D is of paramount importance as it influences the VDR expression directly. Vitamin D has an impact on VDR upregulation by increasing VDR synthesis or decreasing receptor degradation (36, 37) Studies showed that vitamin D induced and stabilized the expression of VDR-messenger ribonucleic acid (mRNA) which led to the VDR level increment (38). The nongenomic effect signaling activation through the membrane bund VDR leads to MAP-kinase stimulation and involves interaction with ion channels, crosstalk with the nuclear VDR, and other transduction pathways. The non-genomic pathway provides a rapid response to the stimulus. However, the magnitude of the extracellular VDR effect still needs to be elucidated (39).

The current study showed that the increase in VDR levels is in line with vitamin D levels which is also depicted by the positive correlation between VDR and vitamin D status. It is supported by a previous randomized controlled trial that found vitamin D supplementation (2,000 IU/day) for 2 months increased the VDR gene expression 60 times (*p* = 0.001) (40). However, the VDR upregulation by vitamin D was suggested to be time and tissue-dependent (41). Furthermore, a systematic review and meta-analyses indicated that the response of the amount of vitamin D on VDR might be different in each individual due to genetic polymorphisms. For instance, TaqI and FokI polymorphisms have been associated with better responses to supplementation of vitamin D (42). Genetic, hormonal (PTH hormone, glucocorticoids, retinoic acid), epigenetic, and environmental factors (diet, sun exposure, pollution, and illness) have an impact on the control of VDR expression. Iriani et al. found that calcitriol increased the mRNA levels of the VDR gene (34). The inline progression of VDR and vitamin D level might be related to the negative regulation of vitamin D by membrane VDR. The membrane VDR works as the receptor of the rapid effect of 1,25(OH)_2_D that induces a variety signaling pathways, for instance the pathway of phosphatidylinositol-3-kinase (PI3K), p21ras, Wnt5a, and phospholipase A_2_ tyrosine kinase Src. Extracellular signal-regulated kinase 1/2 (ERK 1/2), PKC, JNK 1/2, PI3K, and p21ras rapid activation has been shown to regulate CYP24A1 induction by 1,25(OH)_2_D (43).

The regulation of CYP2R1 is still poorly understood. There have been several factors linked to the CYP2R1 regulation. Clinical factors, including starvation, obesity, and diabetes, as well as genetic factors, for instance, mutation and polymorphism, were found to influence the expression of CYP2R1 (44). Roizen et al. found that aging significantly reduces the level of CYP2R1 in the liver of male mice, there has been no study indicating the correlation between CYP2R1 and age in pediatrics (45). In this study, age was found to be negatively associated with CYP2R1. The strength of the correlation test in both variables was weak, indicating the possibility of intermediate variables, for instance, vitamin D. This study also showed that vitamin D levels were lower in older children. Calcitriol suppression induced the expression of CYP2R1 mRNA in oral squamous cell carcinoma tumor cells. In the same study, treatment with calcitriol increased the expression of CYP2R1, proving the relationship between vitamin D and CYP2R1 level (46).

The two cytochromes, CYP2R1 and CYP24A1, pose a contracting effect on vitamin D levels. As CYP2R1 activates calcitriol, the CYP24A1 works as the catabolic enzyme (47, 48). Interestingly, a positive correlation was found between CYP2R1 and CYP24A1. The metabolism of vitamin D appears to be optimally regulated to prevent the synthesis of excess hormone and to break down the hormone or even its substrate by superinducing catabolic mechanisms such as CYP24A1 when needed (15). CYP24A1 is mainly affected by calcitriol, the active form of vitamin D which is first converted by CYP2R1. Calcitriol is known as a strong upregulation factor of CYP24A1 (44). CYP24A1 is not expressed in the absence of calcitriol. On the contrary, CYP24A1 is abundantly expressed in the kidney when calcitriol is detected to limit calcitriol production (49).

This study is the first to depict the association between vitamin D, VDR, CYP2R1, and CYP24A1 in Indonesian pediatrics. Geography and ethnicity are important factors in vitamin D metabolism as it is related to sun exposure intensity, the amount of melanin, and behavioral factors. In addition, the major strength of the current study is that we exclusively included healthy individuals. It is a sensible strategy to reduce variability by removing the impact of diseases on vitamin D levels, VDR, CYP2R1, and CYP24A1. Nevertheless, the current study used a cross-sectional design which does not reveal the causality between correlated factors. Moreover, this study did not include the status of lactation, diet, vitamin D supplementation, and the length of sun exposure of the subjects. Further studies with longitudinal design and inclusion of other variables are required to overcome this limitation.

## Conclusion

In conclusion, our findings suggest that vitamin D level is reduced by the increase in age which suggests the need for vitamin D screening in children older than 2 years old. Vitamin D supplementation might benefit children in the adolescent age group. VDR level was also found to be significantly different based on Vitamin D status. Increasing vitamin D levels induces upregulation of VDR. Both vitamin D metabolism enzymes, CYP2R1 and CYP24A1, suggested inline-level progression.

## Data availability statement

The raw data supporting the conclusions of this article will be made available by the authors, without undue reservation.

## Ethics statement

The studies involving humans were approved by the Helsinki Declaration by the Ethics Commission of the Faculty of Medicine, YARSI University. The studies were conducted in accordance with the local legislation and institutional requirements. Written informed consent for participation in this study was provided by the participants’ legal guardians/next of kin.

## Author contributions

AI: Conceptualization, Formal analysis, Methodology, Project administration, Resources, Supervision, Validation, Writing – original draft, Writing – review & editing. AR: Conceptualization, Methodology, Supervision, Validation, Writing – original draft, Writing – review & editing. MF: Formal analysis, Investigation, Writing – original draft. RG: Formal analysis, Investigation, Writing – original draft. AT: Formal analysis, Investigation, Writing – original draft. FM: Formal analysis, Visualization, Writing – original draft, Writing – review & editing. MN: Funding acquisition, Project administration, Resources, Writing – original draft.
